# Hirayama’s Disease in a Young Male: A Rare Case Report

**DOI:** 10.7759/cureus.6204

**Published:** 2019-11-19

**Authors:** Mukesh Kumar, Pal Satyajit Singh Athwal, Sandeep Rhandhawa, Sukhmanii Kahlon, Jeevan Shiv Kumar

**Affiliations:** 1 Neurology, Shaheed Mohtarma Benazir Bhutto Medical University, Larkana, PAK; 2 Internal Medicine, Saraswathi Institute of Medical Sciences, Hapur, IND; 3 Internal Medicine, University of Hawaii, Hawaii, USA; 4 Internal Medicine, Medical University of the Americas, Camps, KNA; 5 Internal Medicine, J.J.M. Medical College, Davanagere, IND

**Keywords:** hirayama disease, juvenile non-progressive amyotrophy., monomelic amyotrophy

## Abstract

Hirayama disease is a rare neurological condition also known as monomelic amyotrophy (MMA). It is a type of cervical myelopathy, which involves the anterior horn cells and affects the distal upper extremities. It is self-limited, asymmetrical lower motor weakness of hands and forearms. Young males are more commonly affected. The condition is hypothesized to occur due to an asymmetric compression of the cervical spinal cord by the dural sac, however, the exact mechanism(s) continue to be investigated. We report a case of a 20-year-old male who presented with complaints of right hand and forearm weakness, who was diagnosed with Hirayama disease and treated.

## Introduction

Keizo Hirayama described this condition as juvenile muscular atrophy of unilateral upper extremity in 1959 [1]. While the term “Monomelic Amyotrophy” was introduced in 1984, to introduce more specificity into the characterization of the disease [2]. The first pathological study was not performed until 1989 [3]. As a motor neuron disorder, it leads to atrophy of the involved muscles of the upper extremity due to imbalance between the vertebral column, and spinal canal content growth makes the dural sac thicker, and these changes displace the posterior dural sac anteriorly on neck flexion, resulting in compression. Sensory and autonomic involvement is rare [4]. Atrophy slowly progresses and reaches a plateau typically over the course of several years before stabilizing. It is more prevalent in Asia with a clear male predominance, particularly in the third decade of life [5]. In India, only 279 cases have been reported over a 35-year period [6].

## Case presentation

**Figure 1:**
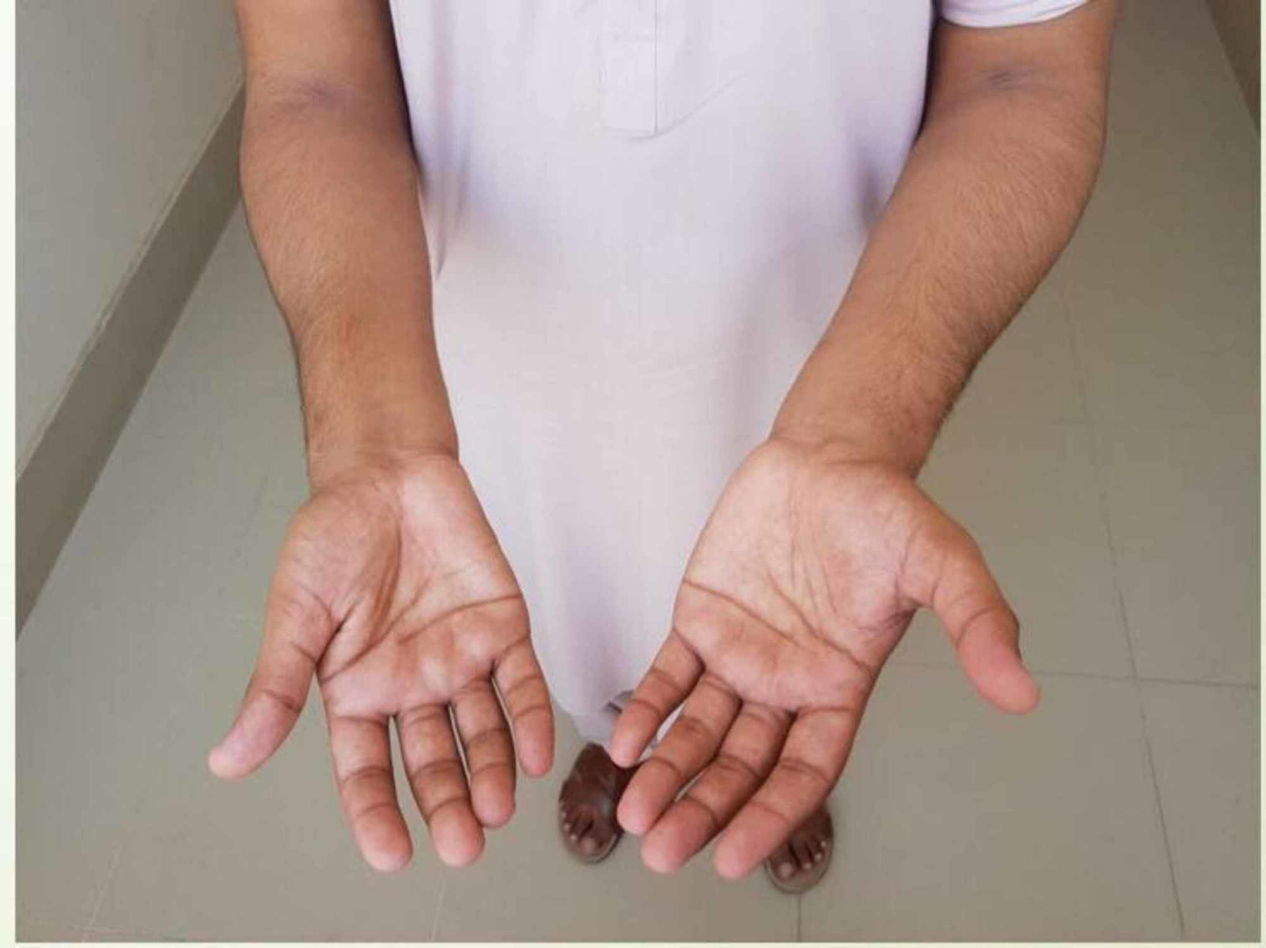
Atrophied right forearm compared to left

**Figure 2:**
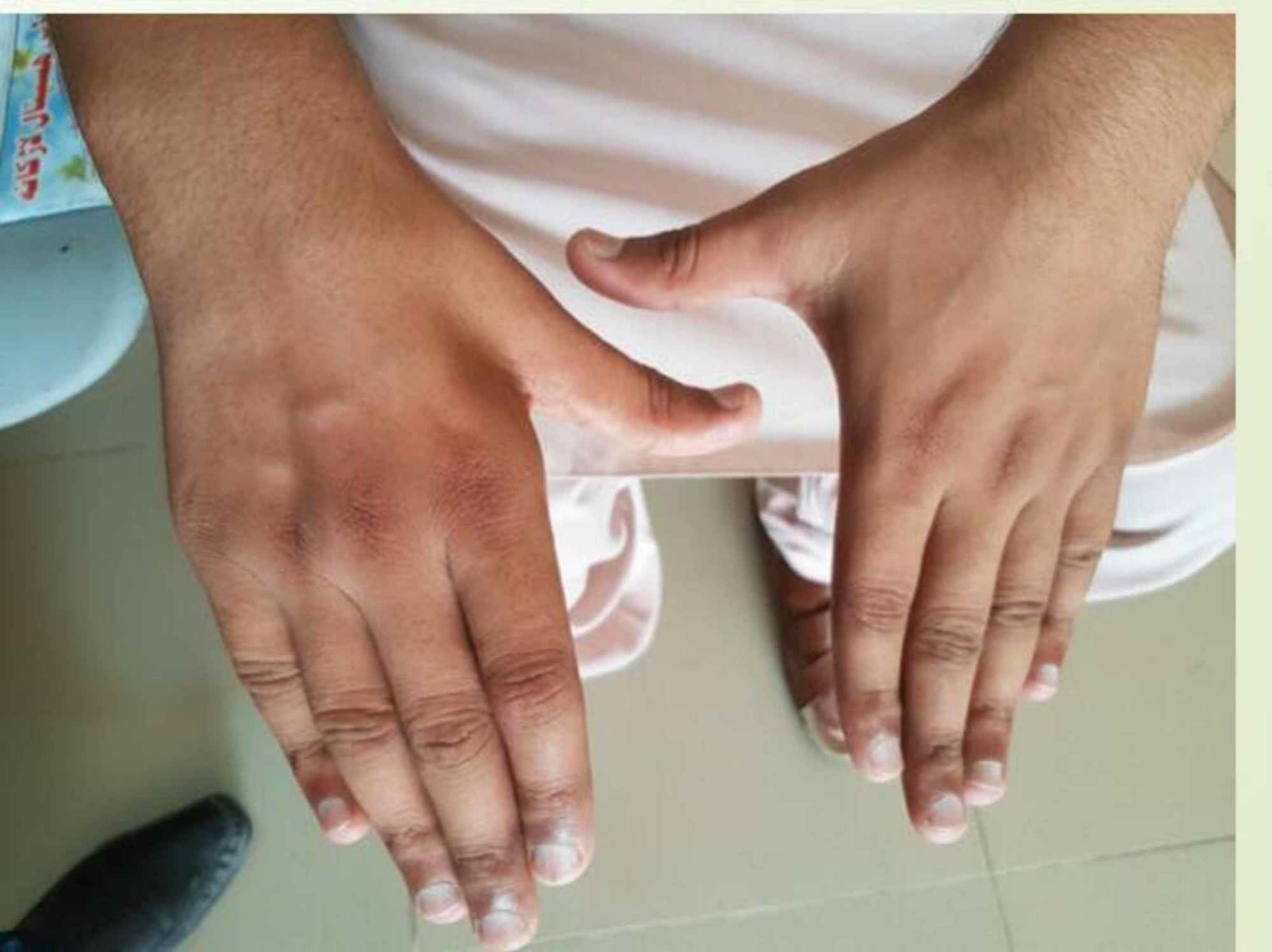
Right hand showing muscular atrophy as compared to the left hand

A 20-year-old male presented with complaints of gradual worsening of right hand and forearm weakness over one year duration. He initially noticed difficulty holding objects with his right hand, but this progressed to difficulty with fine motor skills such as buttoning his shirt and writing. He noticed an insidious decrease in muscle mass of the right hand and forearm. The patient denied any loss of sensation, autonomic dysfunction, bowel or bladder changes, or axial neck pain. There was a noncontributory family history, past medical history and past surgical history, and no history of trauma or prior hospitalization. On examination, muscle bulk was reduced in the right forearm and hand as compared to the left as evident from Figure 1 and Figure 2. The interossei, thenar, hypothenar, flexor and extensor muscles were involved, and the brachioradialis was spared. Tone was slightly reduced at the right wrist with muscle strength of 3/5 in the right hand. Deep tendon reflexes were 2+ bilaterally in the upper limbs. Sensation was intact bilaterally. The plantar reflex was normal. Vital signs were within normal limits.

Nerve conduction studies were done with following results:

Motor: Bilateral median and ulnar nerve conduction revealed normal distal latencies, compound motor action potential (CMAP) amplitudes and conduction velocities. The CMAP amplitudes were relatively lower on the right side. The right ulnar short segmental (inching) study over a 10-cm segment across the elbow revealed evidence of focal demyelination (focal slowing) at the elbow crease.

Sensory: The right median and ulnar nerve conduction studies revealed normal onset latencies, sensory nerve action potentials (SNAP) amplitudes and conduction studies.

EMG/Needle exam: Needle electromyography (EMG) exam revealed evidence of a chronic axonal neurogenic process in bilateral C8-T1 more than C7 innervated musculature, bilaterally along with the mild active denervation in some of the C8-T1 innervated muscles. The process was more severe on the right side.

**Figure 3:**
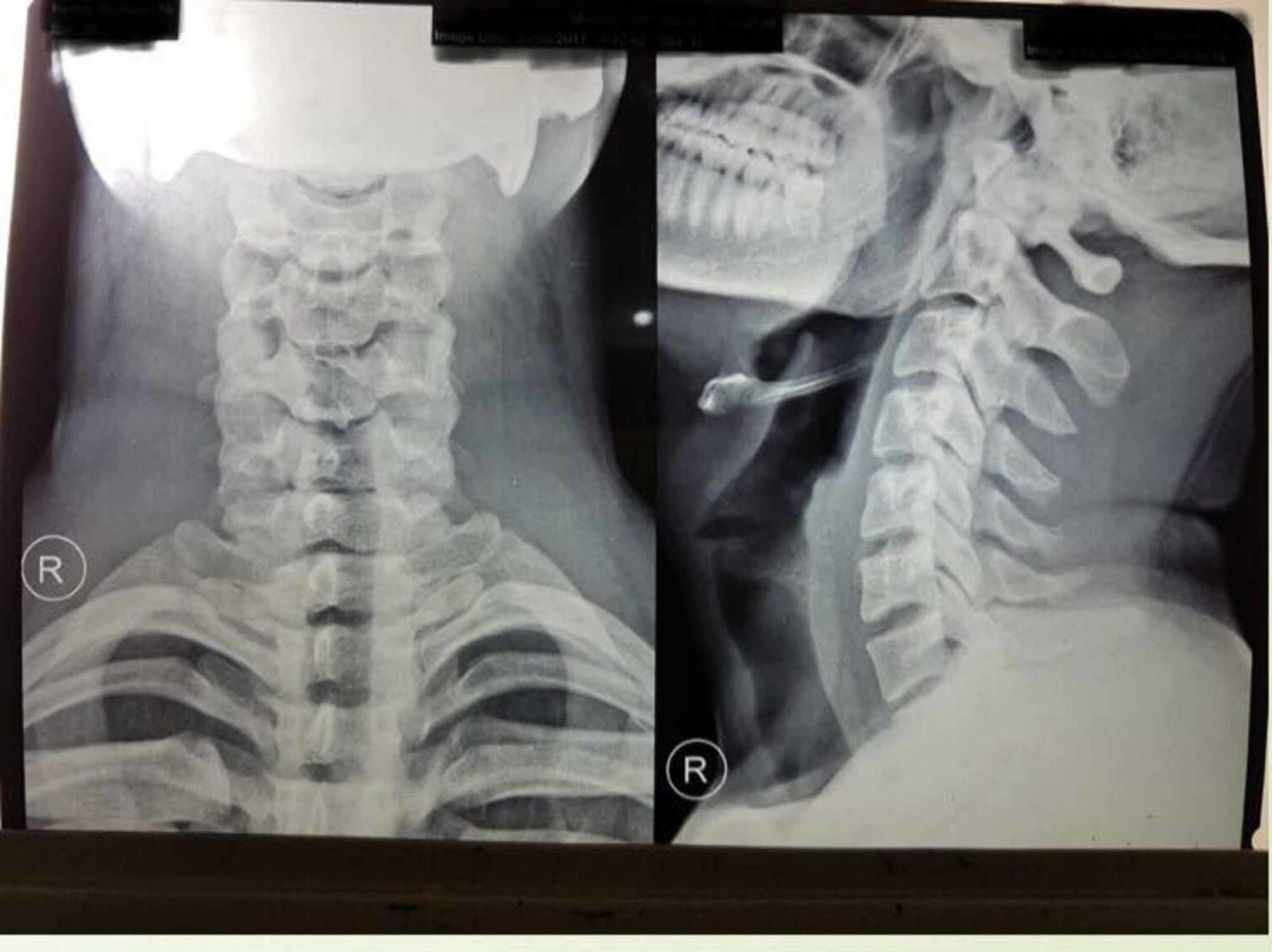
X-ray cervical spine of the patient

Magnetic resonance imaging (MRI) showed a loss of cervical lordosis with C5-C6 and C5-C7 thecal sac indentation. X-rays ruled out any osteophytes or bony deformity (Figure 3). The patient’s complete blood count, kidney function test, and liver function tests were within normal limits as per the standardized lab values. All viral markers including HIV were negative. Hirayama disease was diagnosed based on the history, muscle involvement, nerve conduction studies and MRI findings. The patient was advised to use cervical collar to prevent neck flexion and further compression of the cervical cord.

## Discussion

Monomelic amyotrophy is a rare neurological condition characterized by asymmetrical weakness and wasting of muscles of upper limb, involving predominantly the lower cervical cord C7, C8, and T1 myotomes. Sensory and reflex examination is normal. Autonomic system is involved rarely as reported by Hassan et al. [7]. According to Tashiro et al. [8], the following criteria are important for diagnosis:

(1) Distal predominant muscle weakness and atrophy in the forearm and hand;

(2) Involvement of the unilateral upper extremity in most cases;

(3) Onset between the ages of 10 to early 20s;

(4) Insidious onset with gradual progression for the first several years, followed by stabilization;

(5) No lower extremity involvement;

(6) No sensory disturbance or tendon reflex abnormalities; and

(7) Exclusion of other diseases.

All of these points were fulfilled by our case, which helped make the diagnosis. While pathogenesis remains unknown, it is believed that impaired functioning of anterior horn cells due to pressure atrophy at the level of C5-T1 may play a role [3]. The imbalance between the vertebral column and spinal canal content growth makes the dural sac thicker, and these changes displace the posterior dural sac anteriorly on neck flexion, resulting in compression. Repeated compression or prolonged flexion may lead to ischemic injury to anterior horn cells, which are particularly vulnerable. Cervical X-ray usually shows no changes, as it was the case here. MRI on flexion of the neck typically shows anterior displacement of the posterior dural sac along with a crescent mass in the posterior epidural space of the lower cervical canal [9]. A study conducted by Guo et al. on 14 patients with Hirayama disease showed characteristic segmental injury in the anterior horn cells of the lower cervical cord, while a few patients exhibited extensive neurogenic injury [10]. Other conditions, like demyelinating polyneuropathy in HIV, congenital neuropathies, amyotrophic lateral sclerosis, post-polio syndrome, and syringomyelia should be ruled out by history and appropriate clinical investigation. Diagnosis is made based on clinical and diagnostic findings, and MRI and EMG are often necessary tests. Monomelic amyotrophy is a self-limiting condition without definitive treatment. Cervical collars can be used to prevent the neck flexion, compression and mitigate symptom exacerbation. Apart from permanent cervical fixation, posterior cervical decompression with coagulation of epidural venous plexus is a technique that seems to be effective.

## Conclusions

According to our case report, Hirayama’s disease is a rare type of monomelic amyotrophy neurological condition, characterized by asymmetrical weakness and wasting of muscles of the upper limb, involving predominantly the lower cervical cord C7, C8, T1 myotomes and sparing the autonomic system. The pathophysiology is due to the displacement of the posterior cervical dural sac, which causes spinal cord compression leading to ischemia of the anterior horn cells. This disproportionate growth of the cervical cord and spine usually is seen in adolescence. This case study may be important because if diagnosed early, its progression can be stopped by simple management using cervical collars and can decrease the long-term surgical interventions.
